# Unorthodox cause of urinary leak post radical prostatectomy: Catheter balloon within a bladder diverticulum – Case report and highlights on various methods to overcome leaks

**DOI:** 10.1016/j.ijscr.2020.02.015

**Published:** 2020-02-07

**Authors:** Jad A. Degheili, Haya Malhas, Tag Keun Yoo

**Affiliations:** a Division of Urology, Department of Surgery, American University of Beirut – Medical Center, Riad El-Solh 1107 2020, Beirut, Lebanon; b Department of Medicine, Mubarak Al-Kabeer Hospital, Jabriya, Kuwait city, Al Asimah, Kuwait; c Department of Urology, Nowon Eulji Medical Center, Eulji University School of Medicine, 68, Hangeulbiseok-ro, Nowon-gu, Seoul, South Korea

**Keywords:** Prostatectomy, Prostate cancer, Urinary leak, Diverticulum, Cystography

## Abstract

**Introduction:**

Radical prostatectomy for prostate cancer has been increasingly adopted, especially for localized disease. Nevertheless, this has not been without any morbidities. Complications, such as urine leak, related to malpositioned or malfunctional indwelling catheters, are rarely reported.

**Presentation of case:**

A 75-year-old male underwent an open radical prostatectomy for prostate cancer. Soon after surgery, he witnessed urine leak and extravasation. Imaging including a plain CT scan with a retrograde cystography, showed the tip of the indwelling catheter within a diverticular pouch of the bladder; itself possessing two anterior diverticular pouches, giving the shape of a “Mickey-Mouse” like bladder. The balloon was deflated, and the Foley catheter was repositioned within the bladder, under fluoroscopy. The urine leak from the surgical wound and through the urethra, completely vanished.

**Discussion:**

Urinary leak due to indwelling catheter malfunction or malposition, post radical prostatectomy, is very rare. Only one similar case has been reported so far. Vesicourethral anastomotic leak post prostatectomy is commonly seen, and mostly due to disruption in the posterior anastomosis. Risk factors for such leaks are various and may include: large prostate, previous transurethral resections, techniques adopted for anastomosis and bladder neck reconstruction, among others. Most leakage cases resolve spontaneously or using conservative measures. Those requiring more aggressive interventions are a minority.

**Conclusion:**

Urethrovesical anastomotic leaks are commonly seen after radical prostatectomy. Although most cases are self-limited; others can be managed by various minimally-invasive procedures, diverting urine away from the anastomosis, giving it a chance to properly heal.

## Highlights

• Radical prostatectomy is the surgery of choice for localized prostate cancer.
• Urethrovesical anastomotic leak is uncommon post radical prostatectomy.
• Various minimally invasive procedures are warranted prior surgical re-intervention.
• Malpositioned catheter tip within a bladder diverticulum is an unusual etiology.

## 1 Introduction

Prostate cancer is the most common solid cancer among American men [1]. About 1 in 7 men will be diagnosed with prostate cancer during their lifetime, and usually it is a disease of the elderly population [2]. Prostate cancer is the third leading cause of death following lung and colorectal cancer, respectively [2]. Multimodality treatment approaches for prostate cancer are diverse and include: active surveillance, radical prostatectomy, external beam radiation, androgen deprivation therapy, among others [3].

Radical Prostatectomy is, nevertheless, not without any accompanied complications. Urinary leak post prostatectomy is one of the early complications following such procedure, with reported incidence ranging between 0.3% and 15.4% [4]. The reason behind that is usually multifactorial in origin.

We hereby report a case of urinary leak soon after open retropubic radical prostatectomy, secondary to an incidentally misplaced indwelling catheter within a bladder diverticulum. As far to our knowledge, this is the second case reported in the English literature. This work has been reported in line with the SCARE criteria [5].

## 2 Case presentation

A 75-year-old hypertensive man presented with an elevated PSA of 9 ng/dL and a normal prostate on digital rectal examination. Following a multi-parametric MRI (mp-MRI) of the prostate, a transrectal ultrasound-guided biopsy confirmed a Gleason 6 prostate cancer disease in both lobes, consistent with a cT1c disease. Metastatic work-up was negative. Patient elected to undergo an open radical retropubic prostatectomy. No intraoperative complications were encountered.

Post-operatively, the drainage from the Foley catheter was not as expected, and intravenous fluid was initially increased. The patient was clinically doing well, until we gradually noticed increase in urine leak around the indwelling catheter, which we initially thought secondary to bladder spasms. Later, a soaked wound dressing, having a urine-like odor, was noted. Repeated blood tests revealed leukocytosis of 23,000/μL, with 86% left shift, and an elevated serum creatinine of 2.14 mg/dL, from a 1.14 mg/dL baseline. Urine leak was suspected. Creatinine level taken from the wound drainage was 48.16 mg/dL, which confirmed the diagnosis of a urine leak. Although a malfunctioned indwelling catheter was suspected, irrigation through the catheter confirmed its patency. Broad-spectrum antibiotics were started.

**Fig. 1:**
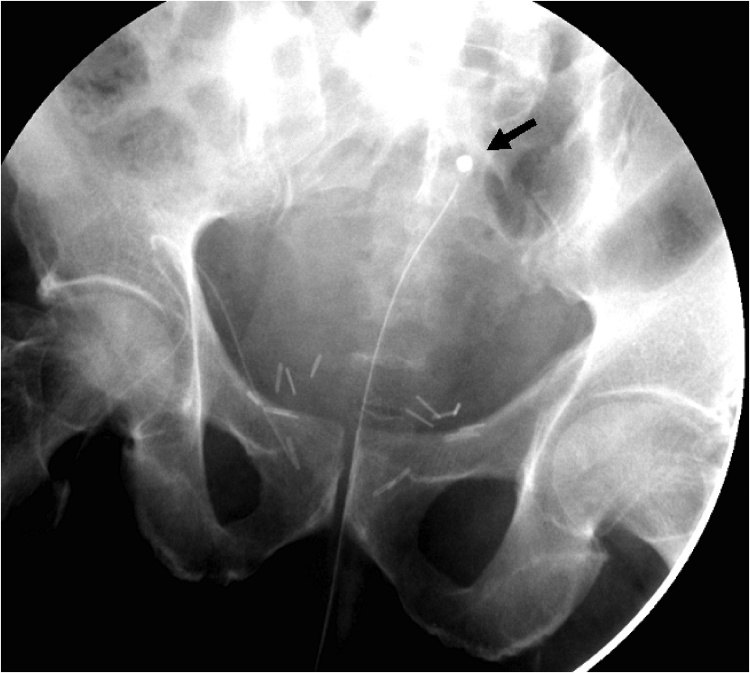
A scout KUB image showing the distal tip of the indwelling catheter at a more cephalad position within the bladder (arrow), at the level of the sacroiliac joint, suggestive most likely to be located within a bladder diverticulum. Note also the surgical clips at the level of the symphysis pubis.

**Fig. 2:**
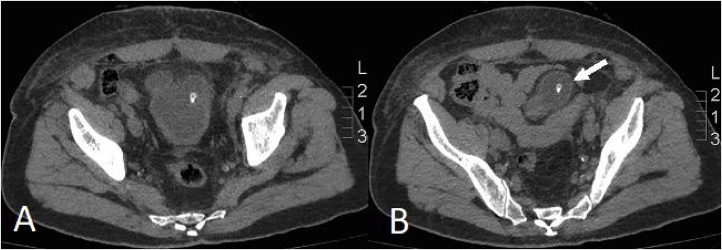
A plain Computed Tomography scan of the pelvis, showing the catheter to be located within the bladder (**A**), and advanced, with the tip seen within a bladder diverticulum (arrow), located at the left anterior bladder wall (**B**).

**Fig. 3:**
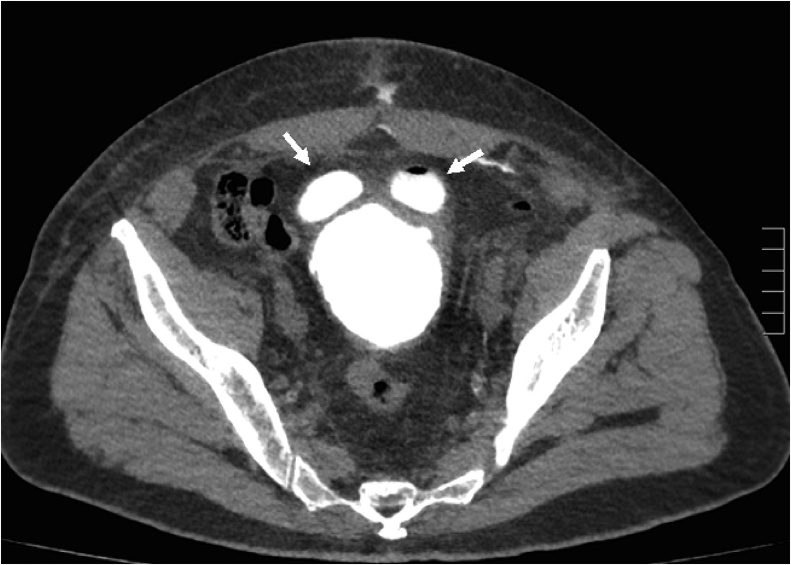
A CT cystography, post filling of the bladder with contrast, showing the presence of two anterior bladder wall diverticula (arrows), rendering the bladder having the “Mickey-Mouse” like appearance.

**Fig. 4:**
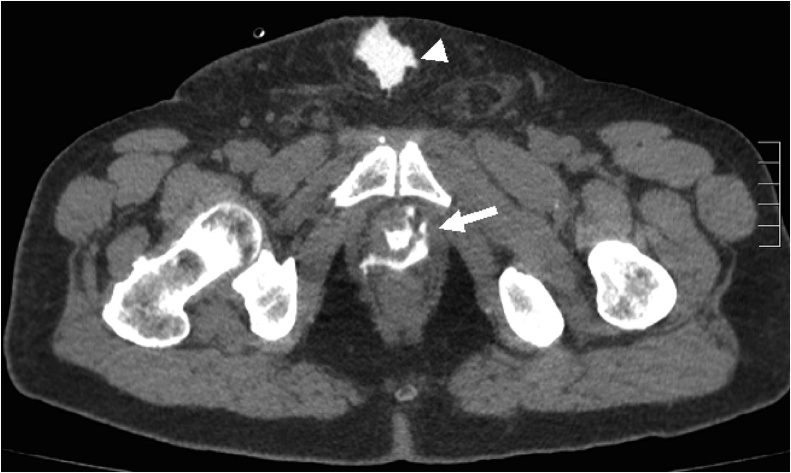
A CT scan showing leakage of contrast at the level of the urethro-vesical anastomosis (arrow), with significant contrast extravasation in the subcutaneous tissue at the midline infraumbilical incision site (arrowhead).

A scout KUB image showed the catheter tip at a more cephalad position than the usual position at the bladder neck (Fig. 1). A plain CT scan was done confirming the tip of the indwelling catheter within a diverticular pouch of the bladder (Fig. 2*A&B*). Retrograde cystography revealed the filling of two anterior diverticular pouches, giving the shape of a “Mickey-Mouse” like bladder (Fig. 3). Leakage at the urethro-vesical anastomosis site was noted, with subsequent seepage of contrast to the subcutaneous tissue and thus from the abdominal wall wound (Fig. 4).

**Fig. 5:**
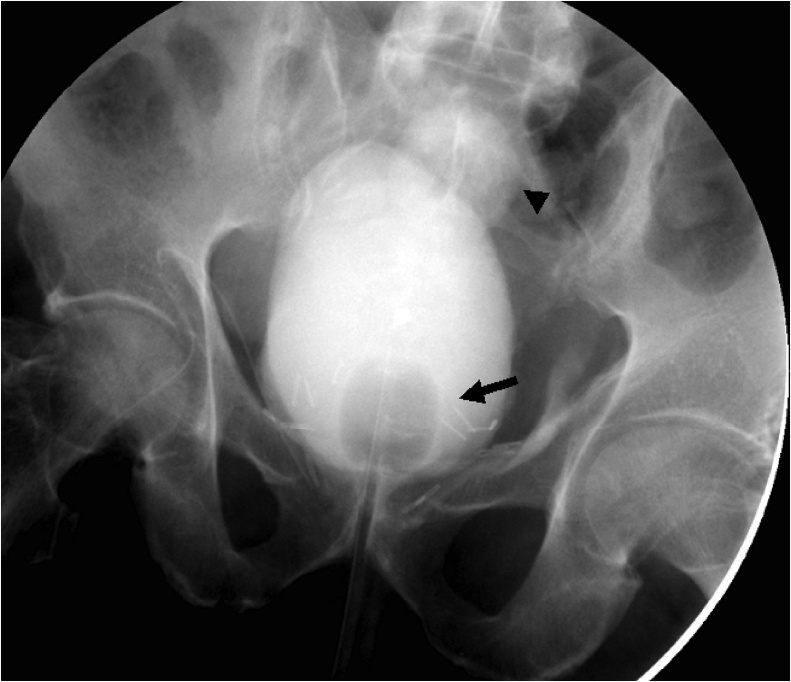
A fluoroscopic image revealing the catheter balloon in good position within the bladder, at the bladder neck (arrow). Note the contour of the anterior left bladder wall diverticulum (arrowhead).

The explanation is that the urine, coming from both ureteral orifices, was not captured by the Foley catheter, which was stuck in a bladder tic, forcing the urine to leak through the urethra-vesicle anastomosis, and from there to the abdominal wound. The balloon was deflated, and the Foley catheter was repositioned within the bladder, under fluoroscopy, and the balloon was again inflated. (Fig. 5). The leak from the surgical wound, and through the urethra, completely stopped.

Follow-up blood tests showed a gradual decline in leucocyte count to 9800/μL and creatinine level to 1.24 mg/dL. The patient was discharged home, four days later. The final pathology was upgraded to Gleason 7(3 + 4) prostate cancer, with negative surgical margins, consistent with pT2cNxMx disease. No lymph nodes dissection was performed.

The indwelling catheter was kept for 2 weeks, and removed after resolution of leak on retrograde cystography. Six months later, the patient was voiding adequately, with no urinary incontinence, and complete wound healing.

## 3 Discussion

Urinary leak due to indwelling catheter malfunction or malposition, post radical prostatectomy, is rare [6], and only one case report, similar to ours, has been reported in the literature [7]. Inadequate drainage of urine from the indwelling catheter can lead to urine leak, given that the urethral-vesical anastomosis has not yet healed, especially directly post-op. Repositioning of the patent catheter within the bladder itself, fluoroscopically, represent the only mainstay management, for leakage to stop. In our case, the tip of the indwelling catheter was located within a bladder diverticulum, and initially were thought of as the cause for urinary leak. Fluoroscopically, this scenario was detected and fixed accordingly.

Complications after a radical prostatectomy are reported between 8% and 18%, including a persistent vesicourethral anastomotic leak in 3.5%–10% of those cases, mostly due to disruption in the posterior anastomosis. Whilst most leakage cases resolve spontaneously or using conservative measures, those requiring more aggressive intervention constitute only 0.9%–2.3% of cases [8].

Several prospective studies from the urology literature have described urinary leak and their various risk factors post radical prostatectomy. Cormio L. et al. highlighted the factors that may lead to urinary leakage after an open radical prostatectomy, including: a large prostate size, low hemoglobin level, low albumin level post-op, previous transurethral resection of the prostate, and the technique adopted for vesicourethral anastomosis and bladder neck reconstruction, especially for large prostate and huge median lobes [9]. Urinary leakage will lead to prolonged ileus, urinoma collection, higher incidences of anastomotic strictures, and incontinence post-op [4]. The incidence of persistent vesicourethral anastomosis after radical prostatectomy has been ranging between 0.9% and 2.5% [10].

Various simplified methods have been utilized when persistent urinary leak is witnessed directly after prostatectomy. It has been postulated that in certain cases with large median lobe, this prompts wider resection at the bladder neck resulting in having the two ureteral orifices in proximity to the resected margin. As such, a posterior bladder neck or “tennis racquet” reconstruction is performed for wide bladder neck. This maneuver result in further approximation of the ureteral orifices toward the median axis. The resultant anatomical changes after reconstruction can result in increase in pressure in this area, the so called “neo-trigone;” thus increasing the risk of urinary leak within the anastomosis itself [11].

Various non-invasive methods have been implemented to overcome urinary leaks. Bhatt et al. replaced the urethral indwelling catheter with a fenestrated 14Fr Pigtail Coop Loop catheter, over a guidewire, permitting intrinsic suction effect through the multiple side fenestrations, with a significant decrease in Jackson-Pratt drainage the following few days [12]. Elmor et al. and Yossepowitch et al. described an endoscopic approach toward treating persistent vesicourethral leakage by inserting bilateral single J ureteral stents measuring 5Fr to 6Fr, and then exteriorized through the urethra, and attached to an indwelling catheter [13,14]. This will direct the urine out via the urethra; reducing the leakage through the fistula and allowing its closure. The ureteral stents were removed around a week later, after confirmation of absence of leakage by retrograde cystogram. The indwelling catheter is kept for three weeks. Both studies reported no bladder neck stenosis or persistent incontinence on long-term follow up [13,14].

Another successful technique to treat leakage was reported by Diamand et al., utilizing a self-made foley catheter, side fenestrated using a Gouge forceps below the level of the balloon, allowing complete reversal in fluid output between the foley catheter and the Jackson-Pratt tube drainage, rendering the anastomosis dry [11]. In a prospective randomized study by Riikonen et al., patients post robotic assisted radical prostatectomy were randomized between those with standard catheters verses those with side-fenestrated catheters. The latter group showed significant decrease in leakage rates at the urethrovesical anastomosis [15]. Shah et al. also managed a delayed anastomotic leak by inserting an ipsilateral nephroureteral stent connected to a suction drainage system. This aided in anastomotic healing by suctioning urine from the bladder and the ipsilateral renal pelvis; thus avoiding the need for bilateral nephrostomies [16]. Floseal® and Tisseel® hemostatic agents were also reported in a single case report in managing anastomotic disruption and persistent bleeding from urethrovesical anastomosis [17]. Another management was an active suction of the prevesical space with negative pressure of 7–12 cmH_2_O, resulting in spontaneous stop of leakage in 7 out of 9 cases post laparoscopic radical prostatectomy [18]. Finally, Lim et al. reported administration of N-butyl-2-cyanoacrylate followed by fibrin glue into the vesicourethral anastomotic gap in 10 patients with massive and prolonged anastomotic urinary leak. Resolution of leak was evident in all cases, and authors concluded that it could be a better alternative to open surgical repair [19]. Failure of resolution of leak with those various non-invasive procedures, will prompt surgical repair as a last resort [10].

## 4 Conclusion

Urethrovesical anastomotic leaks are commonly seen after radical prostatectomy. Although most cases are self-limited; other evidences of anastomotic leak have been managed by various minimally-invasive procedures; by diverting urine away from the anastomosis, giving chance for the urethrovesical defect to properly heal. Failure of those various minimally-invasive approaches will eventually prompt surgical repair. Our peculiar case is second of its kind, with the reason of leak not that usual, and interesting.

## Sources of funding

The authors declare no source of external funding for conducting this manuscript.

## Ethical approval

The study such as this case report was exempted from ethical approval by the Institutional Review Board of the American University of Beirut-Medical Center.

## Consent

Written informed consent was obtained from the patient for publication of this case report and accompanying images. A copy of the written consent is available for review by the Editor-in-Chief of this journal on request.

## Author contribution

Degheili JA and Yoo TK carried out the literature review, acquisition of the radiological images, and wrote the initial draft of the manuscript, incorporating all changes and revisions advised thereafter by the corresponding author. Malhas H assisted in the literature review and the acquisition of radiological images with their corresponding description. All authors agreed on the final version of the manuscript upon submission.

## Registration of research studies

This is a case report not research study.

## Guarantor

Dr. Jad A. Degheili.

## Provenance and peer review

Editorially reviewed, not externally peer-reviewed.

## Declaration of Competing Interest

The authors declare that they have no competing interests.

## References

[1] American Cancer Society (2018). Key Statistics for Prostate Cancer| Prostate Cancer Facts. https://www.cancer.org/cancer/prostate-cancer/about/key-statistics.html.

[2] (2018). Surveillance, Epidemiology, and End Results Program (SEER). https://seer.cancer.gov/statfacts/html/prost.html.

[3] Skolarus T.A., Wolf A.M., Erb N.L., Brooks D.D., Rivers B.M., Underwood W. (2014). American Cancer Society prostate cancer survivorship care guidelines. CA Cancer J. Clin..

[4] Tyritzis S.I., Katafigiotis I., Constantinides C.A. (2012). All you need to know about urethrovesical anastomotic urinary leakage following radical prostatectomy. J. Urol..

[5] Agha R.A., Borrelli M.R., Farwana R., Koshy K., Fowler A.J., Orgill D.P., SCARE Group (2018). The SCARE 2018 statement: updating consensus Surgical CAse REport (SCARE) guidelines. Int. J. Surg..

[6] Dillioglugil O., Leibman B.D., Leibman N.S., Kattan M.W., Rosas A.L., Scardino P.T. (1997). Risk factors for complications and morbidity after radical retropubic prostatectomy. J. Urol..

[7] Bird E.T., Hakim S. (2003). Cannulization of a bladder diverticulum after radical retropubic prostatectomy: an unusual cause of anastomotic leakage with a patent catheter. Urology.

[8] Tewari A., Sooriakumaran P., Bloch D.A., Seshadri-Kreaden U., Hebert A.E., Wiklund P. (2012). Positive surgical margin and perioperative complication rates of primary surgical treatments for prostate cancer: a systematic review and meta-analysis comparing retropubic, laparoscopic, and robotic prostatectomy. Eur. Urol..

[9] Cormio L., Di Fino G., Scavone C., Maroscia D., Mancini V., Ruocco N. (2016). Prognostic factors for anastomotic urinary leakage following retropubic radical prostatectomy and correlation with voiding outcomes. Medicine (Baltimore).

[10] Castillo O.A., Alston C., Sanchez-Salas R. (2009). Persistent vesicourethral anastomotic leak after laparoscopic radical prostatectomy: laparoscopic solution. Urology..

[11] Diamand R., Al Hajj Obeid W., Accarain A., Limani K., Hawaux E., van Velthoven R. (2017). Management of anastomosis leakage post-RALP: a simple trick for a complex situation. Urol. Case Rep..

[12] Bhatt R., Paradis A., Venkatesh R. (2019). Utility of a pigtail cope loop catheter for bladder drainage in treating a large/persistent urethrovesical anastomotic leak following radical prostatectomy. J. Endourol. Case Rep..

[13] Elmor T.R., Rubinstein M., Lima G., Cruz A.C., Pereira C.F., Rubinstein I. (2016). Minimally invasive treatment of vesicourethral leak after laparoscopic radical prostatectomy. Rev. Col. Bras. Cir..

[14] Yossepowitch O., Baniel J. (2010). Persistent vesicourethral anastomotic leak after radical prostatectomy: a novel endoscopic solution. J. Urol..

[15] Riikonen J., Kaipia A., Matikainen M., Koskimäki J., Kylmälä T., Tammela T.L. (2014). Side-fenestrated catheter decreases leakage at the urethrovesical anastomosis after robot-assisted laparoscopic radical prostatectomy. Scand. J. Urol..

[16] Shah G., Vogel F., Moinzadeh A. (2009). Nephroureteral stent on suction for urethrovesical anastomotic leak after robot-assisted laparoscopic radical prostatectomy. Urology.

[17] Paul C.J., Tobert C.M., Tracy C.R. (2017). Novel management of anastomotic disruption and persistent hematuria following robotic prostatectomy: case report and review of the literature. Urol. Case Rep..

[18] Hora M., Stránský P., Klečka J., Trávníček I., Urge T., Eret V. (2013). Managing urine leakage following laparoscopic radical prostatectomy with active suction of the prevesical space. Wideochir. Inne Tech. Maloinwazyjne.

[19] Lim J.H., You D., Jeong I.G., Park H.K., Ahn H., Kim C.S. (2013). Cystoscopic injection of N-butyl-2-cyanoacrylate followed by fibrin glue for the treatment of persistent or massive vesicourethral anastomotic urine leak after radical prostatectomy. Int. J. Urol..

